# Tissue lithography: Microscale dewaxing to enable retrospective studies on formalin-fixed paraffin-embedded (FFPE) tissue sections

**DOI:** 10.1371/journal.pone.0176691

**Published:** 2017-05-11

**Authors:** Julien F. Cors, Aditya Kashyap, Anna Fomitcheva Khartchenko, Peter Schraml, Govind V. Kaigala

**Affiliations:** 1 IBM Research – Zurich, Rüschlikon, Switzerland; 2 Institute of Pathology and Molecular Pathology, University Hospital Zurich, Zurich, Switzerland; Technion Israel Institute of Technology, ISRAEL

## Abstract

We present a new concept, termed tissue lithography (TL), and its implementation which enables retrospective studies on formalin-fixed paraffin-embedded tissue sections. Tissue lithography uses a microfluidic probe to remove microscale areas of the paraffin layer on formalin-fixed paraffin-embedded biopsy samples. Current practices in sample utilization for research and diagnostics require complete deparaffinization of the sample prior to molecular testing. This imposes strong limitations in terms of the number of tests as well as the time when they can be performed on a single sample. Microscale dewaxing lifts these constraints by permitting deprotection of a fraction of a tissue for testing while keeping the remaining of the sample intact for future analysis. After testing, the sample can be sent back to storage instead of being discarded, as is done in standard workflows. We achieve this microscale dewaxing by hydrodynamically confining nanoliter volumes of xylene on top of the sample with a probe head. We demonstrate micrometer-scale, chromogenic and fluorescence-based immunohistochemistry against multiple biomarkers (p53, CD45, HER2 and β-actin) on tonsil and breast tissue sections and microarrays. We achieve stain patterns as small as 100 μm × 50 μm as well as multiplexed immunostaining within a single tissue microarray core with a 20-fold time reduction for local dewaxing as compared to standard protocols. We also demonstrate a 10-fold reduction in the rehydration time, leading to lower processing times between different stains. We further show the potential of TL for retrospective studies by sequentially dewaxing and staining four individual cores within the same tissue microarray over four consecutive days. By combining tissue lithography with the concept of micro-immunohistochemistry, we implement each step of the IHC protocol—dewaxing, rehydration and staining—with the same microfluidic probe head. Tissue lithography brings a new level of versatility and flexibility in sample processing and budgeting in biobanks, which may alleviate current sample limitations for retrospective studies in biomarker discovery and drug screening.

## Introduction

Biopsy samples are routinely collected from patients for diagnostic and prognostic purposes [1]. With patient or family consent, these samples are collected and stored in different repositories, depending on the type of sample (liquid, solid, cells) and used for research. The role of such repositories, also called “biobanks”, is to provide biological specimens for conducting studies for research in areas pertaining to biomarker discovery [2], genomics [3] and drug development [4], for example. Long-term storage of biological samples is central to retrospective studies, where there is typically a gap of several years between sample collection and sample utilization. In pathology institutes, formalin fixation and paraffin embedding (FFPE) of tissue samples is the most commonly used approach for storing biological specimens, which preserves the tissue morphology as well as the molecular content [5]. In the USA alone, it is estimated that around 300 million tissue samples were stored in tissue archives or tissue biobanks at the beginning of the 2000’s and that this number increases by about 20 million each year [6]. This growing quantity of samples is mostly due to regulations that require samples to be conserved for a period typically of 10 years for diagnostic purposes. Despite this large number of stored samples, a study by Masset *et al*. reported that, among cancer researchers, almost 40% have difficulties obtaining sufficient numbers of biological samples for testing purposes [7]. This paradox can be explained from different perspectives, the first of which is organizational. Biorepositories are not centralized, which implies that researchers might not have access to a specific set of samples, for instance, if samples from a rare disease are required; if they are stored in a repository that is privately held, or if they are linked to another institution. Further, there is a high level of disparity in terms of quality standards in all samples stored. Research studies need high-quality samples, in sufficient numbers and with sufficient tissue to perform the tests, but the rarer the disease, the smaller the amount of sample available [8]. Regulations indeed require most biopsy specimens to be stored for future diagnostic purposes, and therefore such specimens are not always available for research. Finally, tissue samples from biopsies are getting smaller owing to the optimization of screening and therapeutic treatments, whereas multiple tests, such as hematoxylin and eosin (H&E) staining, immunohistochemistry (IHC) and fluorescence *in-situ* hybridization need to be performed. Currently only one such test per section can be realized. There is therefore a clear need for bioanalytical methods that reduce the amount of sample required to perform molecular tests.

Different techniques have been developed to allow accurate and efficient use of samples, such as laser capture microdissection [9], layered IHC [10] and various microfluidic implementations [11,12]. Our team recently developed and implemented the concept of micro-immunohistochemistry (μIHC) in which picoliters of antibody solutions are localized on specific areas of a sample, thus allowing multiplexed testing and abating cross-contamination [13]. Micrometer-scale processing of tissue sections enables a more precise and conservative use of samples. In relation to this, we coined the term “tissue microprocessing” (TMP) for the processing and consumption of small amounts of tissue, but that concept goes beyond the phenotype and also encompasses ideas and methods to locally look at genomic and transcriptomic signatures [14,15]. Central to TMP is the micrometer-scale localization of processing liquid on top of a sample. However, existing TMP implementations require the removal of the protective paraffin layer on the section prior to processing. This implies that all tests must be performed simultaneously or sequentially in a short period of time, and subsequently the sample is archived. This process flow might be acceptable in the context of routine pathological investigations, wherein multiple tests are performed simultaneously. However, in the context of retrospective studies, where the availability of biomaterial to be studied might be limited, timing of the use of a sample becomes critical. Researchers currently need to decide whether to test a sample “now” or to preserve the precious sample for future analysis once new sets of hypothesis have been generated.

**Fig 1:**
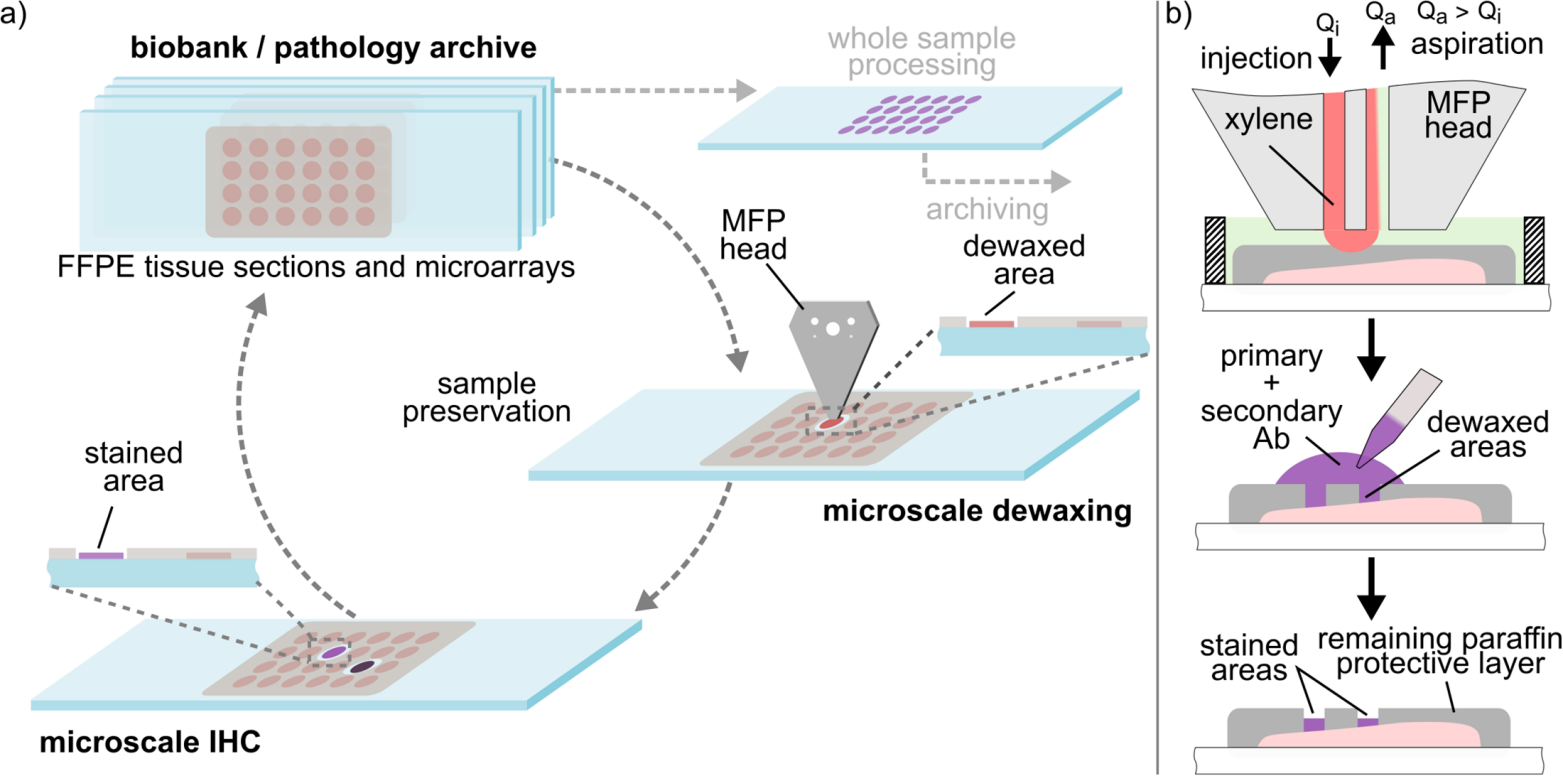
Concept for retrospective studies/diagnostics using tissue lithography. (a) Proposed strategy towards retrospective analysis of archival tissue sections. Microscale dewaxing can be performed on selected areas, enabling subsequent IHC staining of specific areas. The sample can then be stored again in the biobank/pathology archive for future analysis. (b) Tissue lithography on FFPE tissue sections using a microfluidic probe.

In this paper, we propose a new concept and its implementation, called tissue lithography (TL), that allows the removal of small areas of the paraffin layer of FFPE tissue sections, while keeping the remaining tissue intact and protected for future analysis (Fig 1a). We envision that sample preservation by TL will primarily enable a wide range of retrospective studies on biological specimens, particularly on (superfluous) empty tissue and tissue microarray sections provided for both diagnostics and research. TL eases the constraints regarding tissue budgeting by allowing a sample to be returned to the repository after testing rather than having to discard it. Here we implement TL using a scanning probe device—a microfluidic probe (MFP)–to locally remove and create micrometer-scale patterns on the paraffin layer of FFPE tissue sections by hydrodynamically confining nanoliters of xylene (Fig 1b). The remaining paraffin acts as a protective and masking layer, and allows molecular tests such as IHC to be performed on selected areas of the sample. The MFP is a scanning, non-contact, microfluidic-based technology that allows the micrometer-accurate localization of reagents within a wet environment [16]. It relies on hydrodynamic flow confinement (HFC) of a processing liquid within an immersion liquid. To demonstrate the spatial resolution of TL, we performed chromogenic detection of CD45 using diaminobenzidine (DAB) on tonsil tissue sections and microarrays. We show the selective and partial dewaxing of single cores within a microarray as well as the generation of patterns as small as 50 μm × 100 μm. Moreover, we show the versatility of TL by patterning an array of 2 mm × 2 mm on a tissue section, thus showing its larger-scale interaction capabilities. The results we present here suggest the utility if TL for retrospective analysis, with the ability to send a sample back to the repository after use as well as the possibility of performing multiple tests on a single section.

## Materials and methods

### Tissue samples sources

All tissue samples used in this study were purchased through commercial vendors or obtained from a biobank. Tonsil tissue sections and microarrays were obtained from the Tissue Biobank of the University Hospital Zurich. The breast tissue microarrays were purchased through Amsbio (UK).

### Microfluidic probe platform

The MFP platform has four main components: a positioning system, a microfluidic probe head, syringe pumps for flow generation, and a visualization system. The MFP positioning system consists of three linear motorized stages (T-LSM50A-S, Zaber Technologies Inc., Canada). Two of the motorized stages control the X-Y positioning of the sample relative to the MFP head. The third stage controls the distance between the head and the substrate. A custom-made sample holder has been machined and is mounted on the motorized stages. The positioning system is placed on an inverted microscope (Eclipse TI-E, Nikon, Japan) for real-time monitoring during operation. An LED lamp (SOLA, Lumencore Inc., Beaverton, OR) is used for illumination during image acquisition. The head holder consists of a manual dovetail (MT Compact Dovetail, Newport Corp., CA) and a goniometer (Mini-Prism Table, ILEE AG, Switzerland) to ensure parallelism between the substrate and the head apex. A complete description of all components of the compact MFP can be found elsewhere [17].

### MFP head fabrication

The MFP head is a hybrid silicon-glass device comprising six inlets and four apertures at the apex. Three of the inlets are merged using two T-junctions before reaching the apex (see S1 File). The MFP head also comprises a serpentine channel mixing zone to homogenize the solution, resulting in the merging of the three connected inlets. The microchannels were etched in silicon using photolithography combined with deep reactive-ion etching, and are 50 μm × 50 μm in size at the apex. Microchannels were subsequently sealed using anodic bonding (1.3 kV, 480°C). The microfabrication steps are detailed in our previous work [16].

### Tissue lithography on FFPE samples

A PDMS gasket was placed around the paraffin-embedded tissue and filled with a solution of 100% ethanol (immersion liquid). For microscale dewaxing, only two channels in the MFP head are required (one for injection, the other for aspiration) for the confinement of xylene. Tubings, syringes and microchannels were primed with 100% ethanol. Subsequently, the injection channel was filled with xylene. To prevent xylene leakage into the immersion liquid, the aspiration was turned on (flow rate: 10 μl/min) as soon as the MFP head was in contact with the immersion liquid. The MFP head was then brought within 30 μm above the area of the sample to be processed. Xylene injection was started at a flow rate of 2 μl/min. Once the flow confinement had stabilized, the aspiration was reduced to 7 μl/min. After processing the sample, the xylene injection was stopped, and the MFP head was moved out of the immersion liquid.

### On-bench IHC

After microscale dewaxing of the tonsil tissue microarray (University Hospital Zurich), samples were rehydrated by sequential dipping into a series of baths, for 3 min each: 95% ethanol, 75% ethanol, 50% ethanol and tap water. Antigens were retrieved in citrate buffer solution (Target Retrieval Solution, Agilent, Santa Clara, CA) at 37°C for 25 min and blocked with 1% BSA in phosphate-buffered saline (PBS) solution at room temperature for 30 min. A solution of 5 μg/ml of primary antibody (CD45) in PBS was incubated for 25 min at room temperature. Detection of the primary antibody was performed using a DAB/HRP IHC Detection Kit (Abcam, Cambridge, UK).

### Micro-immunohistochemistry (μIHC)

We performed μIHC on partially dewaxed cores of a tissue microarray (NovusBio, CO) using a hierarchical HFC (hHFC). Monoclonal antibodies (Abcam, UK) against HER2 (raised in rabbit) and p53 (raised in mouse) were diluted in PBS with 1% BSA down to a concentration of 25 μg/ml. For priming the tubings as well as for the entire staining procedure, ~250 μl antibody solution was used. The antibody solution was confined (inner confinement) with the MFP on top of the dewaxed areas. To avoid contamination of the immersion liquid with antibody solution and for visualization, an outer confinement with a solution of 10 μM Rhodamine B in PBS was used. During operation, the flow rates used in the four channels were 0.5 and 5 μl/min for injection and –5 and –11.5 μl/min for aspiration. Areas to be patterned were selected based on visual inspection of the tissue. The MFP head was positioned on top of the region of interest, and the antibody solution was incubated for 5 min. The sample was kept immersed in PBS to prevent desiccation.

### Continuous tissue rehydration with the MFP

The three inner injection ports were connected to syringe pumps (neMESYS, Cetoni GmbH, Germany) filled with xylene, ethanol, and water, respectively. The aspiration flow rate was set to 8 μl/min and the injection flow rate to 2 μl/min throughout the experiment. When starting rehydration, only ethanol was injected at 2 μl/min. The ethanol injection was then gradually ramped down to 0, while simultaneously the water injection was ramped up to 2 μl/min. This ensured a gradual and continuous transition between the two solutions in the flow confinement (see S2 Video).

### Image analysis

ImageJ2 was used for image processing. Background subtraction and histogram adjustment were used on each channel. After each individual channel had been processed, the channels were merged. Because the images are used for visualization, some of the processes involve a local increase of the contrast. Images used for direct visual comparison had the same contrast parameters. For image-based quantification, a different algorithm will have to be implemented.

## Results and discussion

### Spatial resolution of TL

**Fig 2:**
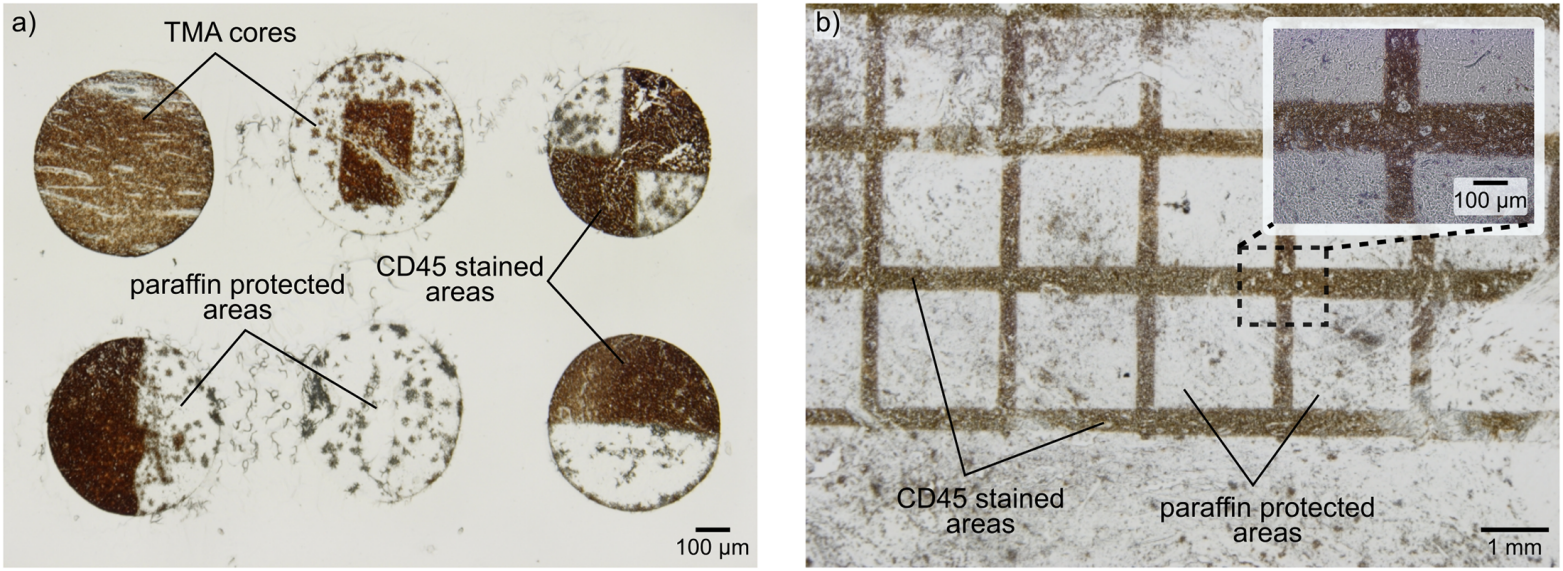
Spatial resolution and patterning capabilities of tissue lithography on FFPE samples. (a) Tissue lithography on a tonsil tissue microarray with chromogenic detection of CD45 proteins using diaminobenzidine. Partial staining of TMA cores highlights the protective effect of the remaining paraffin layer. (b) Large-scale tissue lithography on a tonsil tissue section.

Tissue lithography implemented with the MFP allows local processing and continuous rehydration of FFPE tissue sections and tissue microarrays by removing micrometer-scale areas of the protective paraffin layer, thus leaving the remaining of the sample untouched for future analysis. To demonstrate the spatial resolution of TL as well as larger scale interactions, we performed chromogenic detection of CD45 (transmembrane protein) using diaminobenzidine (DAB) on a tonsil tissue microarray and tissue sections. We were able to selectively dewax single cores (diameter: 600 μm) within a tissue microarray as well as create patterns as small as ~50 μm within a single core (Fig 2a). We further show millimeter-range interactions with TL by patterning an array of 2 mm × 2 squares on the paraffin layer of a tissue section (Fig 2b). After dewaxing, the tissue was rehydrated by sequential dipping of the sample into 95%, 75% and 50% ethanol solutions before a final dip into tap water. Antigen were retrieved using citrate buffer at 37°C for ~25 min. The protective effect of the remaining paraffin layer was verified as the staining is only visible on the dewaxed areas of the sample.

### Fluorescence-based multiplexed IHC

**Fig 3:**
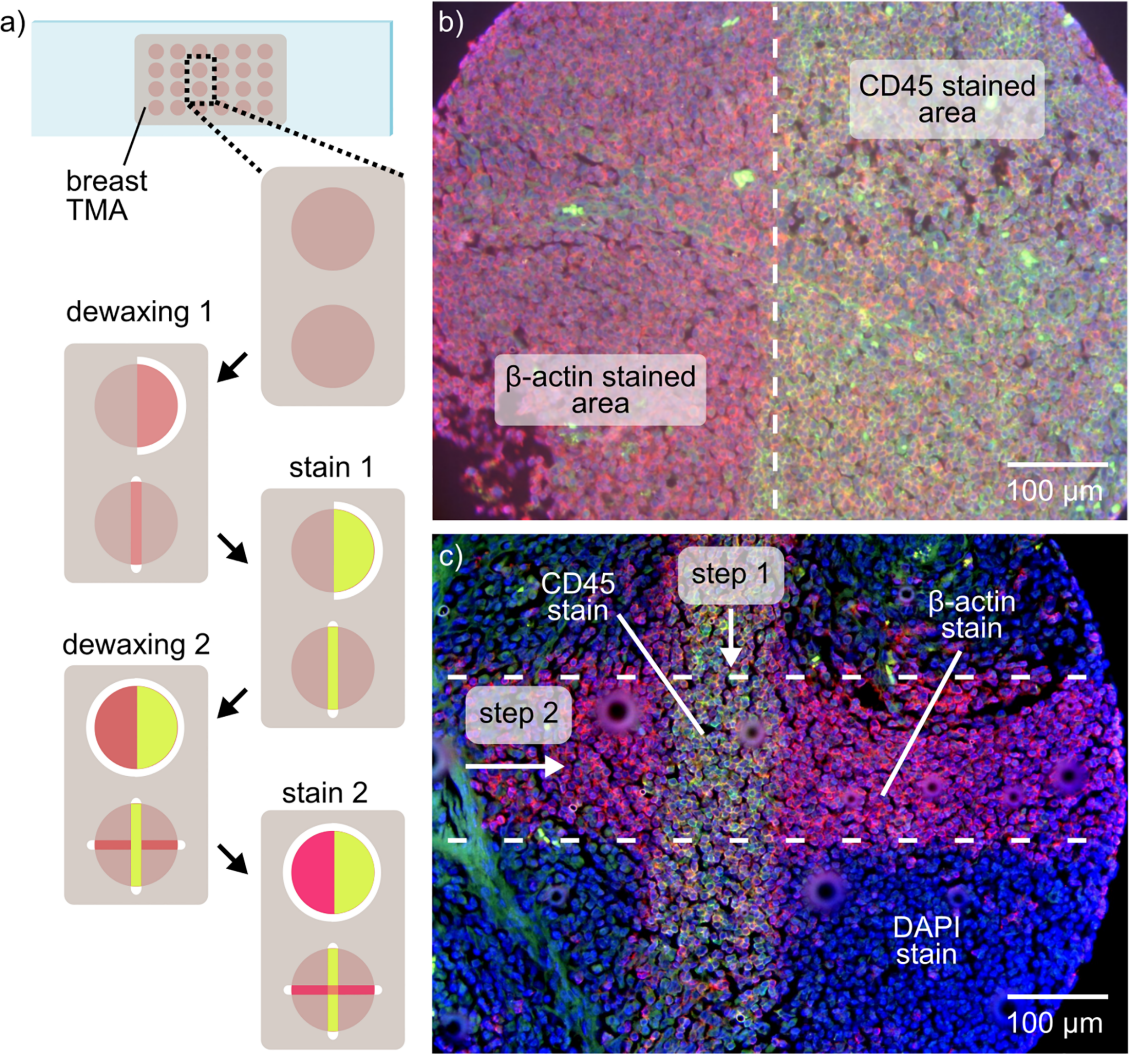
Multiplexed fluorescence-based IHC using tissue lithography. (a) Schematic of the multiple dewaxing and staining steps performed. (b) Overlay image of two fluorescence channels showing multiplexed immunostaining of β-actin (red channel) and CD45 (green channel). (c) Overlay image of three fluorescence channels showing microscale partial staining of a TMA core. The tissue microarray was mounted using DAPI (blue channel) to highlight the non-stained areas of the sample.

A key advantage of TL is the possibility to enable multiplexed micrometer-scale immunostaining by performing multiple sequential microscale dewaxing steps. The protective effect of the paraffin layer allows multiple dewaxing and staining steps to be performed sequentially. Here, we show sequential fluorescence-based IHC staining against CD45 and β-actin using TL on tonsil tissue microarrays. The steps of the procedure are depicted in Fig 3a. After a first dewaxing step, we stained specific areas of the samples, and performed image acquisition and signal analysis for CD45. Subsequently, a second dewaxing and staining step enabled the detection of another biomarker, in this case β-actin. Using this technique, we were able to detect two antibodies within a single TMA core. It should be noted that β-actin stain is also visible on the CD-45 stained area. This is due to the sequence of the dewaxing steps: the dewaxed areas in the first step were also exposed to the primary antibody of the second stain. After staining, the remaining paraffin was removed, and the sample was mounted with DAPI. The overlay image of the three fluorescence channels of the mounted sample shows the regions that were stained against CD45 (green), β-actin (red) and nuclei (blue) (Fig 3b and 3c). This procedure is versatile and tunable according to the requirements of the tests and/or the context of the investigation (pathological investigation or biomarker screening, for example). The number of tests that can be performed on a single sample is a function of the sample size, the percentage of tumor cells found on the sample, and of the amount of sample area required to obtain an adequate staining signal. More importantly, the tests do not have to be performed on the same day: As the non-processed areas of the sample remain protected with paraffin, the sample can be stored and used several days or weeks later for additional tests. The compatibility of TL with both chromogenic and fluorescence-based IHC is demonstrated as both signals were only visible on the dewaxed areas of the tissue. The occurrence of non-specific staining is higher in fluorescence-based IHC, and tends to increase as multiple dewaxing and staining steps are performed. However, as shown in Fig 3c, these issues do not lead to ambivalent results. We attribute this non-specific staining to diffusion of antibodies through the paraffin layer. It tends to increase as multiple dewaxing and staining steps are performed.

### Micro-IHC combined with tissue lithography

**Fig 4:**
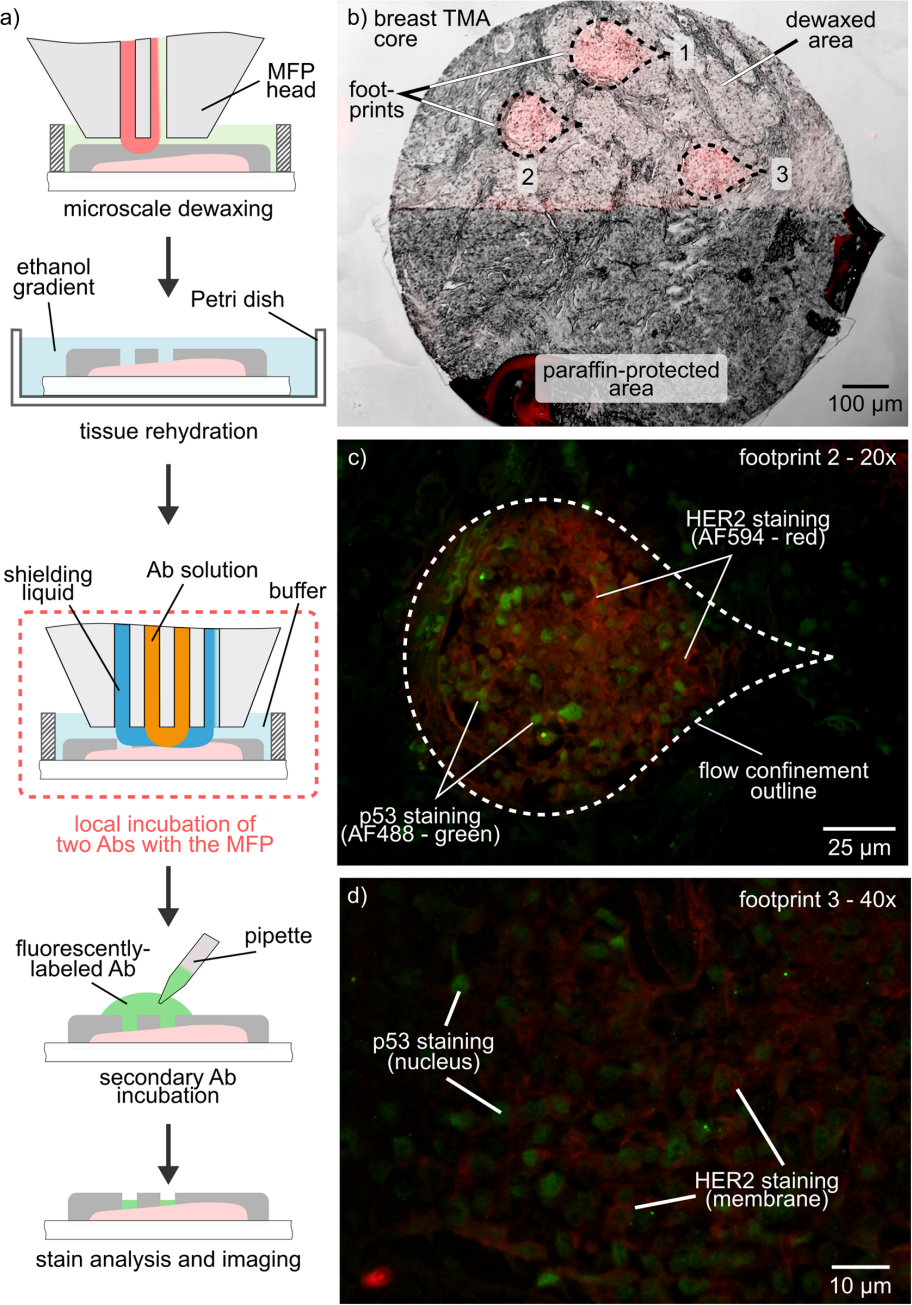
Micrometer-scale co-localization of IHC stains. (a) Schematic of the workflow for localized dewaxing and antibody incubation with the MFP. (b) Overlay image of bright-field and fluorescence (red) images of a breast cancer tissue microarray. Half of the core was dewaxed, and three footprints were incubated with antibodies. (c,d) 20× and 40× images of local HER2-staining (membrane, red channel) and p53 (nucleus, green channel).

To further reduce tissue consumption, we combined TL with our previously published method for performing μIHC. Here, the MFP is used not only to dewax the samples locally, but also to incubate the primary antibody locally within the dewaxed area, thus abating contamination of the remainder of the sample. The workflow of the experiment is shown in Fig 4a. First, TL was performed on a single core of a breast TMA to remove the paraffin on the areas of interest (Fig 4b). Rehydration and antigen retrieval were performed on-bench. We then sequentially incubated three footprints with two primary antibodies against p53 (nuclear marker) and HER2 (membrane marker). The incubation of the primary antibodies was performed using a hierarchical HFC to further reduce the potential risk of leakage of antibodies into the immersion liquid. Finally, the sample was incubated with fluorescently-labeled detection antibodies on-bench, prior to imaging. Fig 4c and 4d shows the fluorescence microscope images of the specific staining of the two biomarkers confined within the footprint in which the flow confinement was applied. With this combination of TL and μIHC, rare samples, such as the ones stored in biobanks, can be processed locally and safely without risking damaging the remainder of the tissue. Such an approach enables a significant increase in the number and the types of tests that can be performed on a single sample.

### Towards retrospective studies

**Fig 5:**
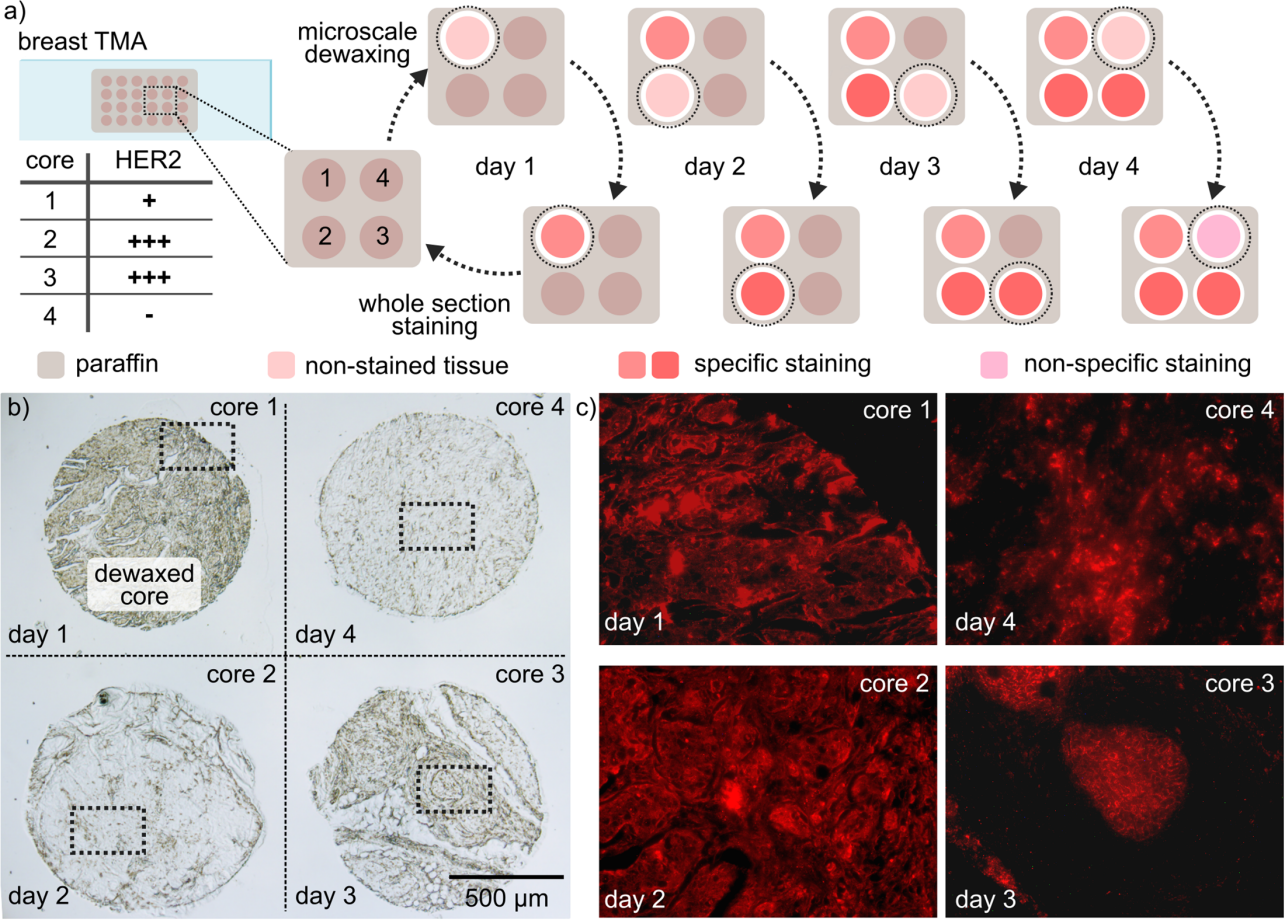
Multi-core staining over multiple days on a single tissue microarray. (a) Schematic workflow of the staining procedure over four days. After each staining step, the sample is stored in the repository, and retrieved on the next day. (b) Bright-field image of the four cores processed during the experiments. The image was taken on day 1 of the experiment, on which core 1 had been dewaxed (darker) and core 2, 3 and 4 were still protected with paraffin. (c) Fluorescence images of the stained cores after dewaxing and staining procedure. Specific HER2 staining is visible on cores 1, 2 and 3, whereas core 4 shows a negative/non-specific stain.

TL enables minimal dewaxing of a tissue section or an array element, which is key to the ability to transport a sample into and out of storage for multiple testing. To demonstrate this, we used TL on four cores of a breast TMA, with each core being processed—dewaxed and stained—on a different day. After microscale dewaxing and on-bench IHC staining, the TMA was sent back to storage and taken out on the next day to process the next core (Fig 5a). Fig 5b shows a bright-field image of the four cores after the first day of the experiment, in which only the first core had been dewaxed. The HER2 staining results obtained for each core correlate with the HER2 status provided in the datasheet of the TMA (Fig 5c). This is the first demonstration of multiple core processing on a single TMA on separate days. We envision the possibility of extending this procedure over several months or years to enable its routine use in retrospective studies. TL would then significantly reduce tissue consumption and ease the constraints on tissue budgeting for retrospective studies.

### Convection-enhanced rehydration

**Fig 6:**
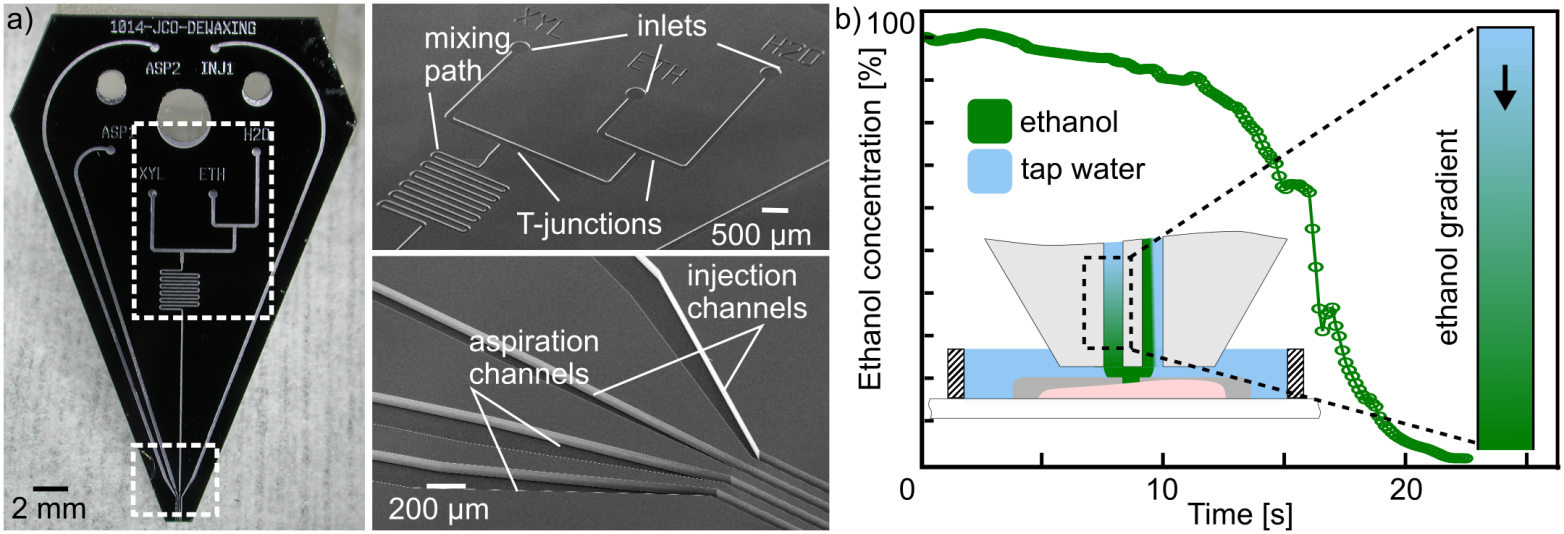
MFP head with multiple inlets and integrated mixer for continuous tissue rehydration. (a) Photograph and close-up SEM images of microfabricated MFP head. (b) Ethanol concentration measured in the flow confinement using fluorescence. A continuous gradient of ethanol to water can be generated to locally rehydrate a sample.

We designed and fabricated a dedicated MFP head that implements all conditions involved in the conventional processing of FFPE tissue sections. This MFP head comprises four apertures to generate an hHFC for confining multiple liquids simultaneously and “shielding” the processing liquid from the immersion liquid. Multiple inlets are connected to the inner injection aperture to allow switching between three processing solutions. Fig 6a shows the T-junctions and a mixing path that ensures homogenization of the liquid, with minimal dead volumes (see S1 File). We demonstrate the capability of this MFP head to generate continuous local gradients of chemicals on immersed surfaces, which is key in tissue rehydration, for example. We achieved time-varying ethanol gradients, changing the composition of the flow confinement from 100% ethanol to 100% H_2_O within 30 s (see S1 Video). To measure the transition between the ethanol and water solutions, we added a fluorescent dye (Rhodamine B) to the ethanol solution and continuously imaged the flow confinement. Fig 6b shows the fluorescence measurement in the flow confinement, which drops down to almost 0% when the ethanol has been completely replaced by water. The standard rehydration protocol used in pathology institutes involves successive dipping of the sample into multiple solvents baths with fixed concentrations (95%, 70%, 50%, and 0% ethanol). In contrast, with the MFP, we can achieve micrometer-scale rehydration of a tissue within 30 s, whereas typically more than 20 min are required for an on-bench protocol. In contrast to the step-wise standard protocol, this enables also the generation of a continuous gradient, which reduces the osmotic shock and shear stress experienced by the sample.

### TL integration into the IHC staining workflow

**Fig 7:**
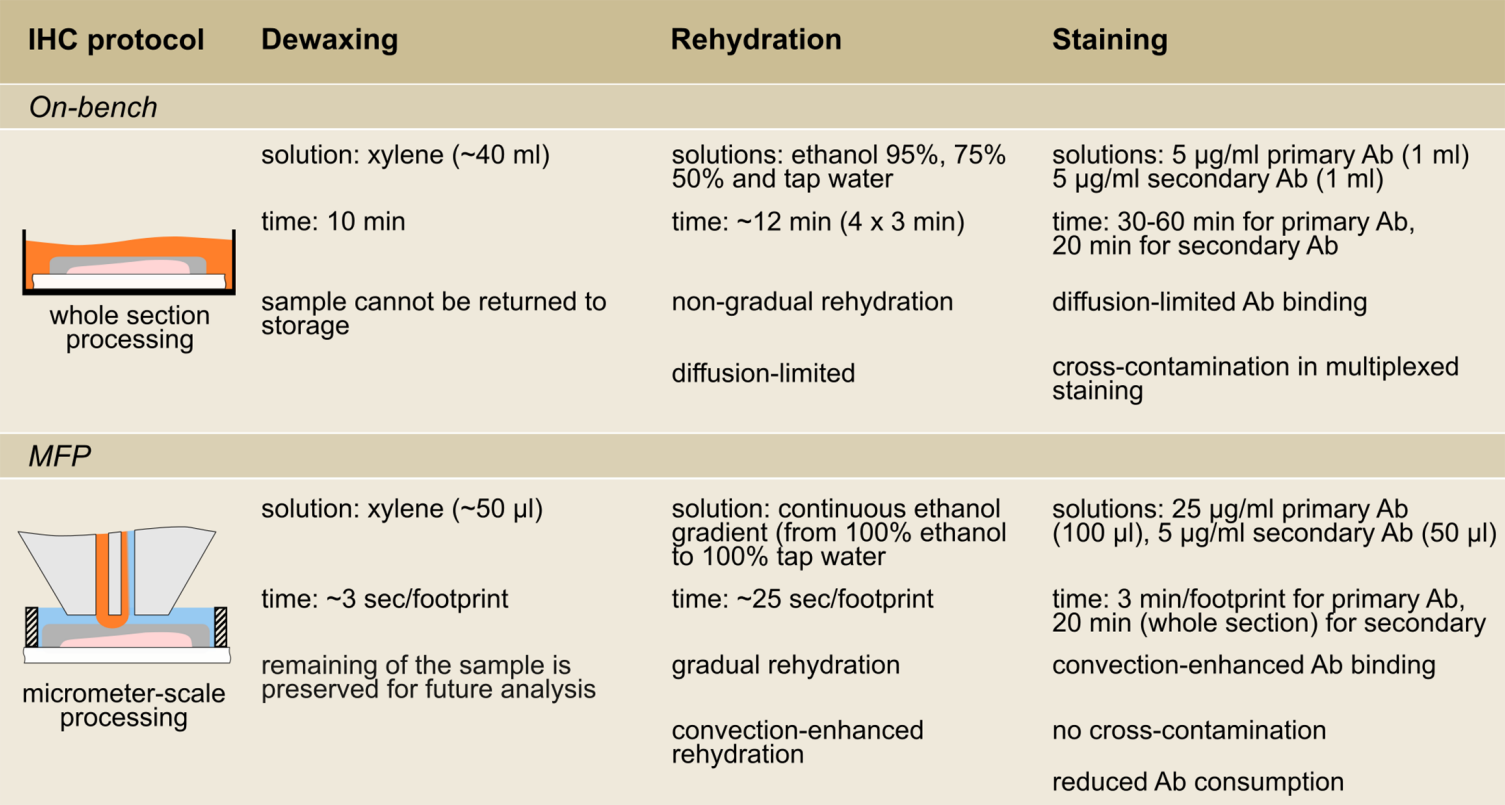
Comparison of on-bench and MFP-based protocol for immunostaining of FFPE samples.

Each result presented in this work addresses one specific aspect related to IHC. The possibility of processing a small fraction of TMA cores as well as larger areas on whole tissue sections provides a high level of versatility in tissue budgeting. Further, the inherent heterogeneity within tissue samples requires a tuning of the processing scale, which is provided by TL. Molecular profiling of a disease requires multiple tests to be performed on several samples from the same patient, and therefore a high number of samples is needed to obtain the complete profile. Currently, only a subset of biomarkers is tested because of sample limitation. With TL, we demonstrated that multiple test can be performed on the same sample or TMA core by leveraging the protective effect of the remaining paraffin layer. This opens the possibility of performing complete profiling of diseases. Combining TL with μIHC allows fine-tuning and optimization of the staining procedure for each biomarker. Incubation of the primary antibody with the MFP gives precise control over the incubation time and concentration, and microscale dewaxing permits multiple tests to be performed until the optimal staining conditions have been determined. All these elements can be used in the context of retrospective studies to test the validity of new findings or hypotheses with minimal tissue consumption and constraints in terms of timing and optimal staining parameters. Here we demonstrated a first proof-of-concept of how a retrospective study with TL could be implemented: a fraction of a sample is processed at a given time and the sample then sent to storage before being taken out again for another test at a later time. In this work, as a proof-of-concept we demonstrated this over four days. Given adequate sample storage and preservation, we envision that this concept can be applied over months or years. For such long periods, the sample would have to be protected by an additional thicker layer of paraffin to prevent loss of antigenicity. In addition to the localization of processing solutions, MFP implementation of IHC steps exhibits an important time advantage in each step (see Fig 7), which is explained by the convective effect inherent in continuous flows within the HFC.

## Conclusion and outlook

We described, implemented, and characterized a new concept of tissue lithography (TL) for enabling micrometer-scale processing of tissue sections. This method relies on hydrodynamic flow confinement (HFC) generated by a microfluidic probe (MFP) to confine solvents and antibody solutions on an immersed biological substrate. Central to all the results presented here is the first demonstration of microscale dewaxing of FFPE tissue sections. This enables the same sample to be processed multiple times without compromising the integrity of the tissue, allowing further storage of the sample in the biorepository. The effect of convection inherent in continuous flows as used in HFC reduces the processing time of the sample in every step of the IHC protocol—dewaxing, rehydration and antibody incubation—compared with the processing time of the diffusion-limited, standard on-bench protocol. The region to be dewaxed needs to be properly determined to prevent false negative staining. A tissue slide with H&E staining can serve as a guide to localize tumor areas. Using TL with μIHC offers several advantages, such as requiring only few hundred microliters of reagents, whereas otherwise typically several milliliters are required. Further, the combination of TL with μIHC not only allows non-specific staining to be minimized by confining the primary antibody, but also finding of the optimal staining conditions with less tissue consumption. The reduction of non-specific staining was shown by co-localizing two stains (p53 and HER2) on three areas as small as 100 μm × 75 μm. The paraffin was removed on half of the tissue core, leaving the other half available and protected for future tests. A first proof-of-concept for retrospective studies was given as four cores of a TMA were individually dewaxed and stained over the course of four days. To the best of our knowledge, this is the first demonstration of IHC staining on multiple TMA cores spread over multiple days.

TL in its current implementation is limited in throughput because it only allows the processing of a single micrometer-scale area of the sample at a time, which can be time consuming when larger areas must be analyzed. However, we believe this limitation could be addressed by scaling up the dimensions of the MFP and/or by running multiple HFCs in parallel, as previously demonstrated [18]. Also, performing antigen retrieval with the MFP remains elusive as most protocols require temperatures as high as 90°C, whereas paraffin has a softening temperature around 40°C. This imposes the need for micrometer-scale heating with minimal losses in the surroundings. To solve this, we envision combining TL with the concept of hydrodynamic thermal confinement [19]. Finally, there is a need to develop μIHC further to obtain more quantitative data, as required especially in the retrospective analysis of biomarkers.

TL is readily compatible with the current workflows in pathology and has the potential of providing a necessary advancement in biobanking techniques. We also envision the relevance of TL in the context of routine pathological examinations, where the microprocessing of the tissue would allow the transfer of the sample back and forth between a source and a second or a third opinion. We strongly believe that this new concept of TL brings a new level of versatility in sample processing and budgeting, which will abate the current sample limitations for retrospective studies.

## Supporting information

S1 File MFP head design and fabrication. (a) Mask layout of the MFP head for microscale dewaxing and rehydration with T-junctions and a mixing zone. (b) Microfabrication protocol of the MFP head using photolithography.
(DOCX)

S1 Video Video of microscale dewaxing. Accelerated video (6×) of microscale dewaxing (10× magnification) of 600 μm diameter tonsil TMA core.
(MP4)

S2 Video Video of ethanol gradient generation in the flow confinement. Real-time video of a continuous transition from 100% ethanol to 100% water in the flow confinement.
(MP4)
