# Supplementary material for: Tissue lithography: Microscale dewaxing to enable retrospective studies on formalin-fixed paraffin-embedded (FFPE) tissue sections
Source: PLoS One. 2017 May 11;12(5):e0176691. doi: 10.1371/journal.pone.0176691 (PMC5426611; doi:10.1371/journal.pone.0176691)
Supplement: S1 File — (a) Mask layout of the MFP head for microscale dewaxing and rehydration with T-junctions and a mixing zone. (b) Microfabrication protocol of the MFP head using photolithography. (DOCX) [file pone.0176691.s001.docx]

Supporting Information

**Tissue Lithography: Microscale Dewaxing to Enable Retrospective Studies on Formalin-Fixed Paraffin Embedded (FFPE) Tissue Sections**

Julien F. Cors^1^, Aditya Kashyap^1^, Anna Fomitcheva Khartchenko^1^, Peter Schraml^2^ and Govind V. Kaigala^1^*

^1^ IBM Research – Zurich, Säumerstrasse 4, 8803 Rüschlikon, Switzerland

^2^ Universitätsspital Zürich, Rämistrasse 100, 8091 Zurich, Switzerland

* Corresponding author

E-mail: gov@zurich.ibm.com

**S1 - MFP Head design and fabrication**

**
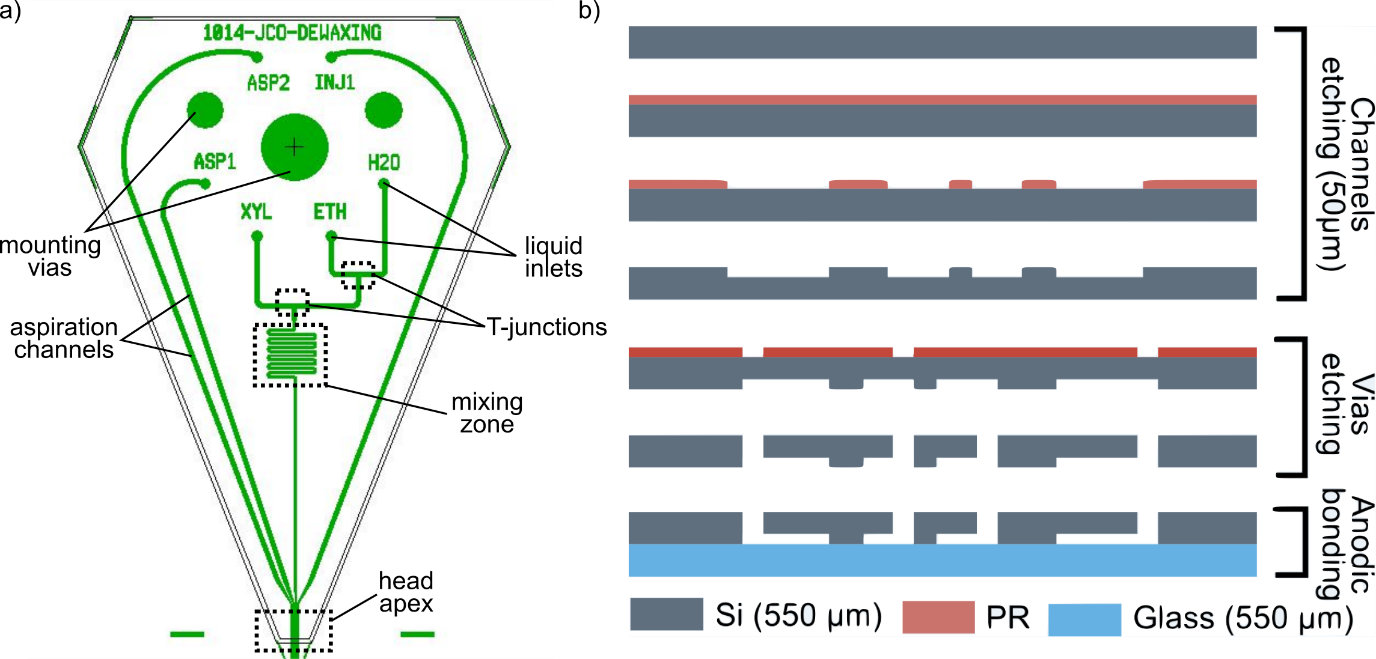
Fig S1. MFP head design and fabrication.** (a) Mask layout of the MFP head for microscale dewaxing and rehydration with T-junctions and a mixing zone. (b) Microfabrication protocol of the MFP head using photolithography.

When performing rehydration, a continuous transition between ethanol and water is required in the flow confinement. Because of the laminar properties of the flows in microchannels, a longer flow path is required for the two phases to be completely mixing. The length of the flow path is determined using the Fourier number theory to Fick’s second law of diffusion:

$$\begin{aligned} {Fo}_{m}=\frac{Dt_{r}}{D_{h}^{2}} \#\left( S1 \right) \end{aligned}$$

Physically, the higher the Fourier number, the more optimal the mixing of the two phases.


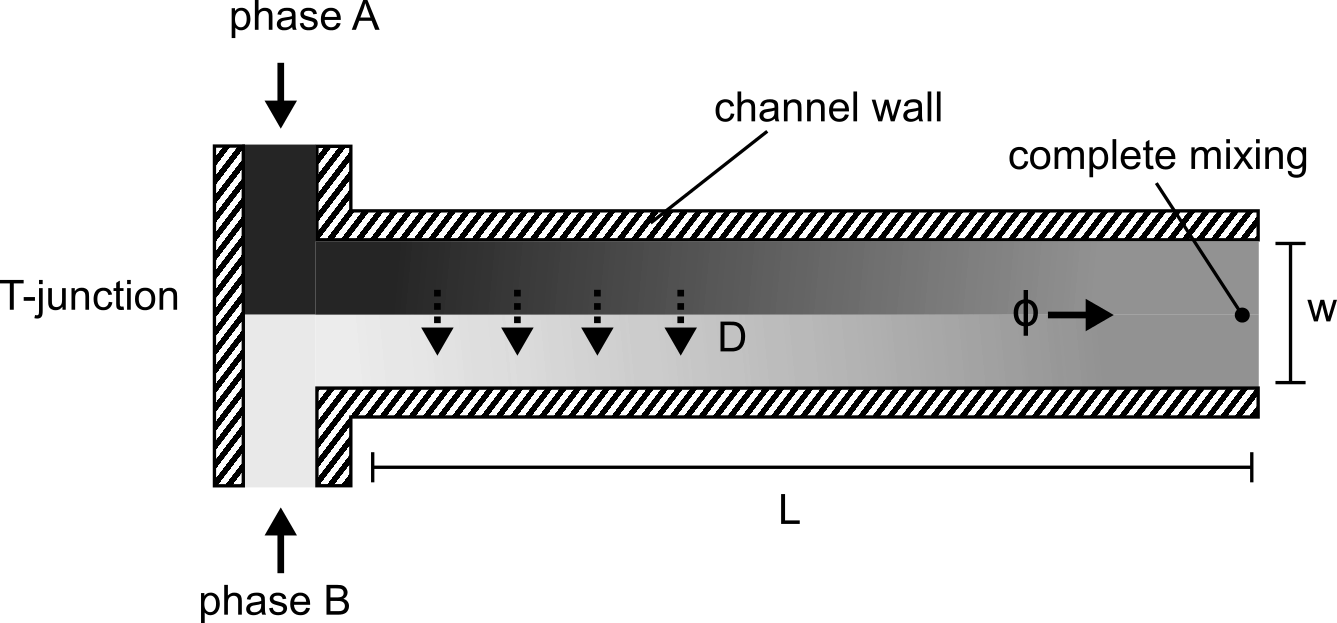


**Fig S2. Schematic of analytical model of mixing between two parallel laminar flows inside a microchannel.**

For a rectangular channel, the characteristic length is given by:

$$\begin{aligned} D_{h}=\frac{2wh}{w+h} \#\left( S2 \right) \end{aligned}$$

With *w,* the channel width and *h* the channel height.

The residence time in the channel is given by:

$$\begin{aligned} t_{r}=\frac{L}{v}\#\left( S3 \right) \end{aligned}$$

With *L*, the channel length and *v* the fluid velocity.

Assuming complete mixing of the two phases (*Fo = 1*) and substituting S3 into S1, we obtain:

$$\begin{aligned} L=\frac{D_{h}^{2}v}{D}\#\left( S4 \right) \end{aligned}$$

With,

$$\begin{aligned} v= \frac{\Phi}{wh}\#\left( S5 \right) \end{aligned}$$

Where $\Phi$ corresponds to the volumetric flow rate inside the channel.

Assuming $\Phi=2 {\mu l}/{min}$, $D=1.2\cdot{10}^{-9} {m^{2}}/s$, $h=50 \mu m$, and $w=50 \mu m$, we obtain a channel length of $L\cong18 mm$.

With this value, we designed the mixing path to have a length of 30 mm, thus ensuring optimal mixing.
